# Supplementary material for: An Atlas of the Quantitative Protein Expression of Anti-Epileptic-Drug Transporters, Metabolizing Enzymes and Tight Junctions at the Blood–Brain Barrier in Epileptic Patients
Source: Pharmaceutics. 2021 Dec 9;13(12):2122. doi: 10.3390/pharmaceutics13122122 (PMC8708024; doi:10.3390/pharmaceutics13122122)
Supplement: Supplementary file 1 [file pharmaceutics-13-02122-s001.zip › 1465449-supp.pdf]

pharmaceutics

Supplementary Materials: An Atlas of the Quantitative Protein Expression of Anti-Epileptic-Drug Transporters, Metabolizing Enzymes and Tight Junctions at the Blood–Brain Barrier in Epileptic Patients

Risa Sato, Kotaro Ohmori, Mina Umetsu, Masaki Takao, Mitsutoshi Tano, Gerald Grant, Brenda Porter, Anthony Bet, Tetsuya Terasaki and Yasuo Uchida

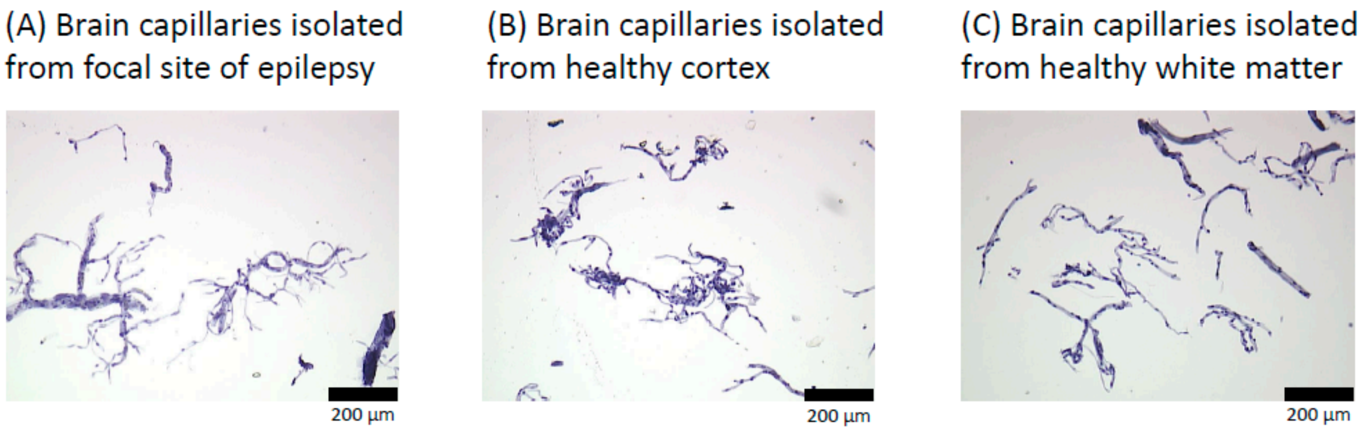

Figure S1. Microscopic photographs of the isolate brain capillaries. (A) Focal siteofepilepticbrain, (B) healthcortex, and (C) healthy whitemat-ter. The scalebar, 200 µm.
